# Evaluation Challenges for the Application of Extended Reality Devices in Medicine

**DOI:** 10.1007/s10278-022-00622-x

**Published:** 2022-04-25

**Authors:** Ryan Beams, Ellenor Brown, Wei-Chung Cheng, Janell S. Joyner, Andrea S. Kim, Kimberly Kontson, Dimitri Amiras, Tassilo Baeuerle, Walter Greenleaf, Rafael J. Grossmann, Atul Gupta, Christoffer Hamilton, Hong Hua, Tran Tu Huynh, Christoph Leuze, Sarah B. Murthi, John Penczek, Jennifer Silva, Brennan Spiegel, Amitabh Varshney, Aldo Badano

**Affiliations:** 1 Center for Devices and Radiological Health, Food and Drug Administration, Silver Spring, MD USA; 2 Department of Imaging, Imperial College Healthcare NHS Trust, London, UK; 3 CognifiSense, Inc., Sunnyvale, CA USA; 4 Stanford University Virtual Human Interaction Lab, Stanford University, Stanford, CA USA; 5 Northern Light Health, Brewer, ME USA; 6 Philips, Cambridge, MA USA; 7 Brainlab AG, Munich, Germany; 8 James C. Wyant College of Optical Sciences, University of Arizona, Tucson, AZ USA; 9 OpticSurg Inc., Wilmington, DE USA; 10 Department of Radiology, Stanford University, Stanford, CA USA; 11 R Adams Cowley Shock Trauma Center, University of Maryland Baltimore, Baltimore, MD USA; 12 NIST, Boulder, CO USA; 13 University of Colorado, Boulder, CO USA; 14 SentiAR, Inc., St Louis, MT USA; 15 School of Medicine, Division of Pediatric Cardiology, Washington University, St Louis, MO USA; 16 Department of Medicine, Cedars-Sinai Medical Center, Los Angeles, CA USA; 17 Department of Computer Science, University of Maryland, College Park, MD USA

**Keywords:** Augmented reality, Virtual reality, Medical imaging, Image quality

## Abstract

Augmented and virtual reality devices are being actively investigated and implemented for a wide range of medical uses. However, significant gaps in the evaluation of these medical devices and applications hinder their regulatory evaluation. Addressing these gaps is critical to demonstrating the devices’ safety and effectiveness. We outline the key technical and clinical evaluation challenges discussed during the US Food and Drug Administration’s public workshop, “Medical Extended Reality: Toward Best Evaluation Practices for Virtual and Augmented Reality in Medicine” and future directions for evaluation method development. Evaluation challenges were categorized into several key technical and clinical areas. Finally, we highlight current efforts in the standards communities and illustrate connections between the evaluation challenges and the intended uses of the medical extended reality (MXR) devices. Participants concluded that additional research is needed to assess the safety and effectiveness of MXR devices across the use cases.

## Background

Medical extended reality (MXR) describes a spectrum of technologies that display virtual objects in the real environment (augmented reality, AR) or present a fully virtual world (virtual reality, VR) for medical applications, often using a head-mounted display (HMD). Interventional procedures and surgery applications are being developed to display virtual medical images and patient-specific anatomical models that can be manipulated and annotated for preoperative planning and registered to the patient for intraoperative navigation [1–3]. HMDs also may be used to capture and share images and video from the surgeon’s perspective for educational use, documentation, or to facilitate real-time guidance and collaboration with a remote surgeon [4]. In addition, novel clinical applications provide engaging environments and training for pain management [5, 6] and psychotherapy [7], and the gamification of physical therapy can be employed to increase patient compliance [8].

Despite recent advances, MXR devices and applications continue to face persistent technological and usability limitations and a lack of standardized evaluation methodologies. Adoption of MXR devices and their integration into the medical workflow require development of evaluation methodologies that quantify the current limitations to ensure safety and effectiveness. Here, we outline the key evaluation challenges discussed during the US Food and Drug Administration’s (FDA) public workshop entitled, “Medical Extended Reality: Toward Best Evaluation Practices for Virtual and Augmented Reality in Medicine,” which took place on March 5, 2020. Finally, we provide examples of evaluation challenges for particular use cases and discuss future directions to address current gaps.

## Methods

The purpose of the workshop was to discuss evaluation techniques for hardware and software, and to identify the critical evaluation gaps that impede the development of safe and effective MXR uses. It was organized into technical and clinical sections with open panel discussions. These sections featured such experts as device manufacturers, academics, medical professionals, medical device companies, and government scientists. Following the workshop, the publicly available recorders were reviewed and the key evaluation challenges were identified. The organizers, moderators, and panelist wrote and reviewed multiple drafts of the consensus statement until a consensus was reached. The key evaluation challenge categories and example use cases discussed during the workshop are summarized in this article.

## Challenges

A MXR system acts as the interface between the human user and the physical and digital worlds. The three-dimensional physical world surrounding the user, visually perceived in real time, can be modeled as a visible light field. The digital world contains information for the user, such as medical data or images acquired from other medical devices; including real, physical world data from these devices, such as external sensors for patient and medical tool tracking.

**Fig. 1:**
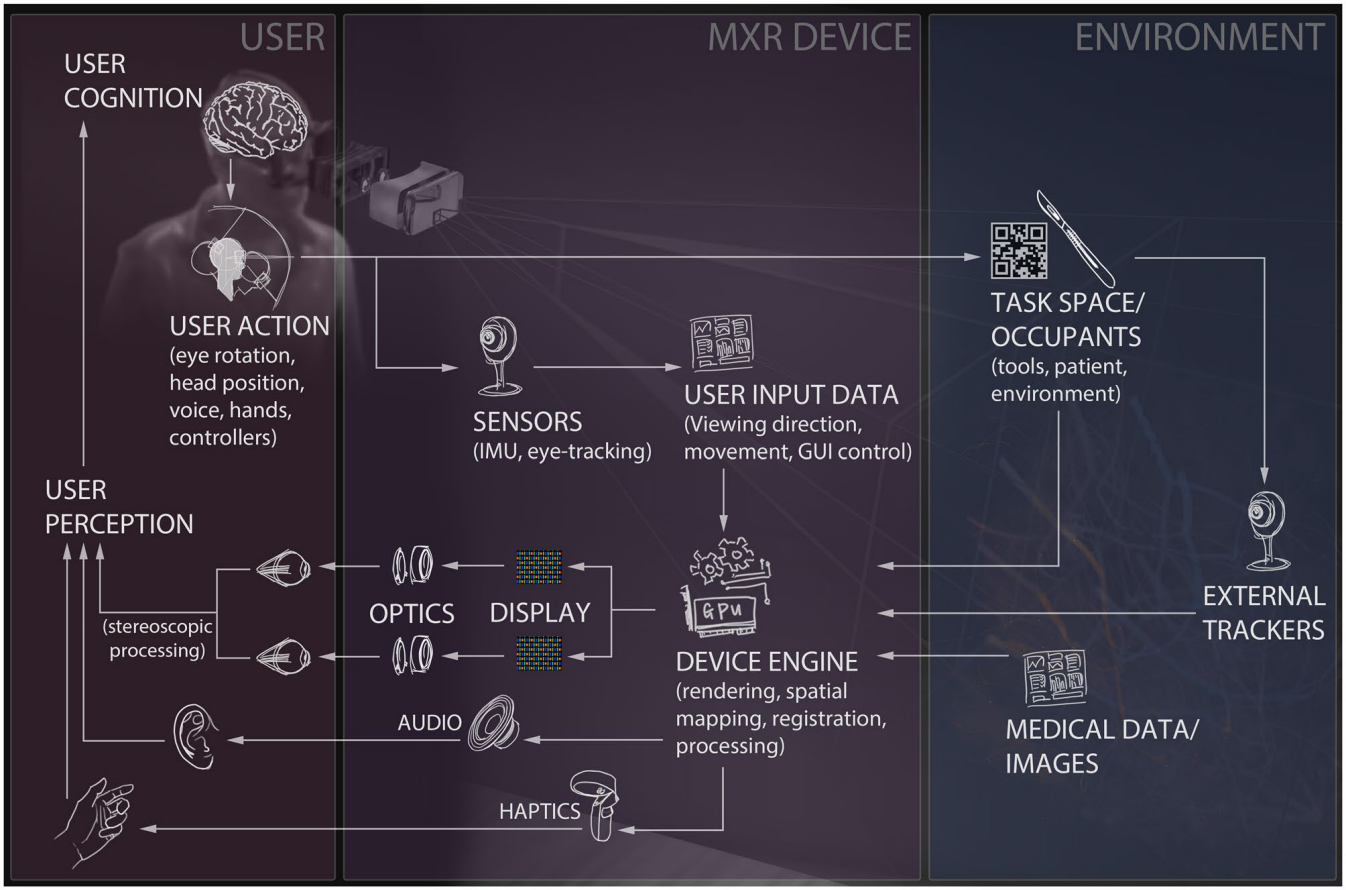
The medical extended reality technology landscape encompasses a wide range of components and connectivity paths between the user, the device hardware and the use environment

Digital data can be dynamically retrieved via wired or wireless networks, or pre-loaded onto the MXR system. The MXR system interacts with the user in various domains from input and output streams. In the output direction from the MXR system to the user, the MXR system delivers optical signals through the display, audio signals through the headset speakers, and mechanical signals through haptic devices. The MXR system also employs various sensors for detecting user input, such as handheld controllers, gesture sensors, microphones, gaze trackers, body movement trackers, or brain wave electroencephalographic sensors. In addition, MXR systems pose challenges related to user comfort, including weight, pressure, and heating caused by the head-mounted device. As illustrated in Fig. 1, interconnection between the data, optics, electronics, and mechanics is critical for creating the desired interface between the user and the physical world. These connections provide the conceptual framework for understanding the interlinked technical and clinical evaluation challenges. In the following subsections, we discuss the key evaluation challenges associated with each component.

### Technical Evaluation

In order to realize the roles of extended reality (XR) medical devices in revolutionizing healthcare, key performance issues need to be addressed. As with any new healthcare technology, innovations in engineering raise new evaluation questions for determining potential improvements in performance or workflow. In the case of XR, engineering and evaluation challenges continue to evolve, as they require consideration regarding device performance and user integration. The first two workshop sessions introduced performance issues, presented current efforts to address these issues, and discussed evaluation challenges for current and future solutions. The key technical evaluation challenges can be categorized in aspects related to image quality and usability. The technical challenges related to these categories are summarized in Table 1.

**Table 1:** Summary table of technical evaluation challenges for MXR applications

**Spatial Image Quality**	**Temporal Image Quality**	**Usability**
Spatial resolution (lateral/depth)	Image latency	Device user interface design
Luminance/Contrast	Motion-to-photon latency	Visual comfort (VAC)
Color performance	User/object movement	Physical ergonomics
Image uniformity across FOV	Latency compensation	Depth perception
Optical aberrations	Image registration accuracy	Scale/location of data
Digital image integrity	Sensor/Tracking accuracy	Impact of physical environment
Rendering artifacts	Rendering artifacts	Cybersickness

#### Image Quality

Evaluation of image quality is particularly challenging for MXR devices due to their diversity and constantly evolving technological characteristics. Image quality encompasses a large set of parameters; including luminance, contrast, temporal and spatial resolution, field of view, dynamic range, frame rate, refresh rate, latency, transmission, and optical aberrations [3, 9–12]. Each aspect of image quality presents unique considerations, and the most suitable testing methodology frequently depends on the specific hardware technology [13]. The evaluation also should consider the varying image quality across a wide field of view [9]. Evaluation challenges are more pronounced in AR due to the ambient lighting, which impacts contrast and color perception. The risks associated with these challenges can be mitigated in some cases by using MXR devices as adjuncts to, instead of as replacements for, standards of care [1].

**Fig. 2:**
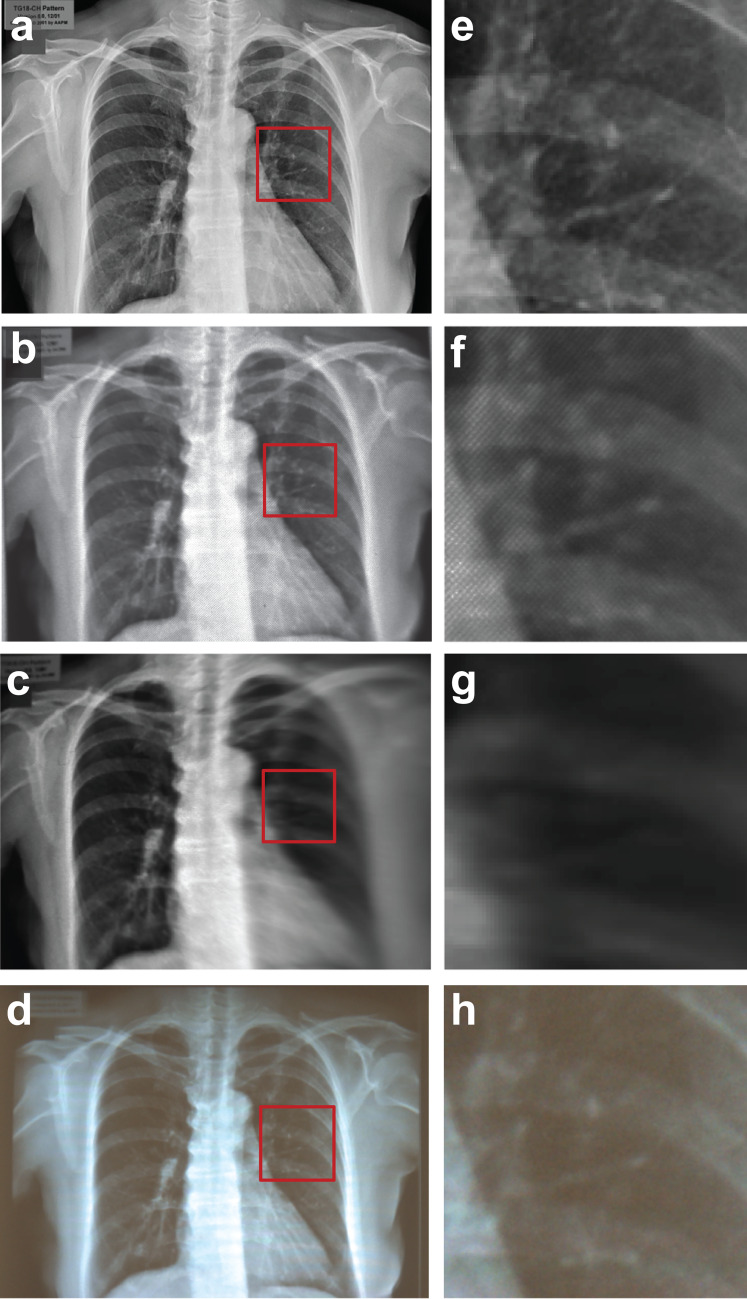
**a**. Input image **b**. Image on VR HMD **c**. Image with incorrect IPD **d**. Image on AR HMD with ambient light. **e**–**h**. Magnified images of the red box in **a**–**d**

Figure 2 illustrates examples of the impact of the HMD on image quality. The initial image sent to the HMD and the resulting image on a VR HMD are shown in Fig. 2a, b, respectively. The image on the VR HMD shows a significant decrease in spatial resolution and contrast compared to the input image. The decrease in image quality become more significant for an interpupillary distance (IPD) that is different than the designed IPD, as shown Fig. 2c, which illustrates both the challenges in image quality and the importance of the usability of the device. Finally, Fig. 2d shows the impact of ambient lighting on image quality for AR HMD. Figure 2e–h shows the region in the red square magnified to more clearly illustrate the changes in image quality in the different conditions.

Beyond the optical and display technology challenges, images viewed within AR and VR environments are subject to temporal errors, [14, 15] as they require real-time updates to account for user and object movement in the environment. Thus, image quality also depends on the status of added systems, such as inertial sensors, user input data, tracking sensor technology, and image registration. For example, registration and superimposition of medical images onto a patient’s anatomy rely on tracking and sensors, as the HMD user and the patient move. Although various motion prediction techniques are utilized for latency compensation, these temporal considerations have performance implications, particularly in image-guided interventional procedures, and currently lack standards.

In addition to image quality considerations from the hardware components, the software and rendering pipeline also introduce unique challenges for MXR devices. MXR devices often utilize commercial game engines for visualization and rendering. The formatting, bit-depth, voxelization, grayscale, and color properties of the input medical images can be impacted by the rendering process due to the use of shaders, material properties, and graphical performance optimization. This is particularly true for diagnostics and surgery planning using radiographic images that generally utilize the Digital Imaging and Communications in Medicine (DICOM) Grayscale Standard Display Function. The impact of these rendering engines on medical image quality is largely unexplored [16], lacking both standards and evaluation methods. Besides the rendering pipeline, formatting of the saved data including biometrics also presents a challenge from both a data standardization perspective and a data security perspective [17].

Image quality and visual ergonomics have been central to the International Electrotechnical Commission (IEC) and International Organization for Standardization (ISO) standards groups on near-eye displays. The IEC has established terminology, measurement geometry, and optical measurement methods to ensure accurate and repeatable results. Current efforts are focused on methodologies for monocular measurements, including contrast, resolution, transmittance, and geometrical distortion [18–20]. The ISO standards have emphasized visual ergonomics, such as visual fatigue or discomfort caused by interocular performance differences [21, 22]. Current ISO standards have gaps around evaluation methods for binocular performance, latency, comprehensive visual guidelines, and new HMD technologies. Similarly, MXR standards have not described the processing of medical images and DICOM data to create 3D render-ready virtual objects. Some MXR applications could benefit from optical and perceptual testing standards, conceptually similar to test procedures developed by the American Association of Physicists in Medicine (AAPM) for medical displays. Standards development for MXR is particularly important because developers frequently use off-the-shelf hardware without having full control over that hardware, which can introduce unintended variability in image quality and ergonomics.

#### Usability and Human Factors

Usability of MXR devices requires that their designs consider the user’s human visual system (HVS) limits of importance for applications for medical professionals and patients. However, designing an HMD that matches the limitations of the HVS presents significant engineering challenges in resolution, field of view, latency, and accurate focal cues. Establishing a quantitative metric for the impact of these parameters on usability is challenging. One example is the location and scale, or size, of virtual content, such as patient vitals or alarms, in the field of view, which impacts the perception across the eccentricities of human vision [23]. A second important example is depth perception and the vergence-accommodation conflict (VAC) caused by the virtual image being at a static distance from the user, while stereoscopic displays give the perception of the object at various depths [11, 12, 24]. The distance of the virtual plane from the user should suit the working distance of the intended application. Most current HMD designs place the virtual image plane at approximately 2 m, which is farther than the working distance for AR tasks within arm’s reach, such as AR-guided surgery with medical images registered to the patient. This mismatch between the virtual and physical objects raises effectiveness questions and evaluation challenges [25, 26]. Optically addressing VAC is an area of research interest [11, 24, 27].

The task and physical environments also impact the usability of MXR devices by adding additional evaluation considerations. For example, a VR device for immersive therapeutics raises different evaluation considerations for usability. Similarly, surgical tasks in interventional suites and operating rooms with bright ambient illumination present unique challenges for the visibility and spatial mapping of AR images, including the visibility of the patient’s anatomy and the virtual medical image overlaid on the patient. In addition to visibility, the perceived accuracy of an image overlaid on a patient also raises usability questions [28]. Different surgical tasks have varying requirements for AR image accuracy, which can influence device design.

Training in the use of MXR devices is another important consideration for the broader community. Providing adequate instruction on the use of a medical device is important, regardless of the level of risk to the user. For those MXR applications associated with higher risks to patient safety (e.g., surgery or interventional procedures), training becomes a key component of the device-user interface for risk mitigation. The appropriate length, frequency, content, and format of training for these emerging applications have yet to be established. To reduce use-related adverse events, additional research into the best training approaches for specific applications should be explored.

From a regulatory perspective, the incorporation of human factors principles throughout device design and development is important in order to comply with 21 CFR 820.30 design control regulations [29]. Human factors considerations also should be informed by the application. MXR devices using fully immersive virtual environments or superimposed virtual objects in real-world environments can lead to visual fatigue, motion sickness, ergonomics concerns, or cognitive overload [30]. Methods by which users control certain features of the MXR devices (e.g., gaze, voice, and gesture) also can influence the user’s experience. The combination of an HMD with voice, gaze, and gesture controls can enable medical professionals to keep their attention and hands on the patient during procedures rather than shifting focus away from the patient to see a monitor or press a button. Understanding the impact of the technology on the end user is an integral step in identifying use-related risks associated with specific applications of these emerging technologies. Standards for human factors assessment, specifically in XR settings involving cybersickness, are in development by ISO, IEC, and Institute of Electrical and Electronics Engineers (IEEE).

#### User and Environment Tracking

Another critical technical performance aspect is the tracking of the user and the environment, including surgical tools, the HMD, and the patient. The accuracy, latency, and smoothness of the tracking impact task performance and affect the accurate rendering of medical images from the user’s perspective to real-time tool visualization in interventional and surgical procedures. Calibration sequences also are necessary to ensure the tools, and HMD is integrated into the use environment and are maintaining the desired performance [31–33]. The accuracy of the tracking also can be impacted by signal interruption, which can lead to systematic offsets in the position of tools. Finally, HMD devices often incorporate tracking systems and are being explored as alternatives to the standard-of-care stereotaxic systems. However, the accuracy of off-the-shelf systems raises performance questions for sensitive procedures.

### Clinical Evaluation

#### Trial Endpoints

Clinical trial endpoints also present unique challenges for MXR devices. Given their wide range of intended uses, the primary and secondary endpoints used to support effectiveness claims and safety in premarket applications need to be clearly defined for each solution. For example, many AR devices are being investigated in the context of interventional and surgical applications to guide procedures [1], facilitate the visualization of patient anatomy [34], or to register and overlay medical images on the patient [28]. In other instances, XR devices are used as immersive therapy for patients across a range of conditions. The evaluation of these applications requires identifying the outcome measures most appropriate for determining if the MXR device, in conjunction with other medical devices, improves surgeon performance and/or patient outcomes. There are two main types of endpoints commonly used to support regulatory review: patient/clinician-reported outcomes and performance-based outcomes. Due to the broad application space for XR technologies, development of device-specific recommendations identifying potential clinical endpoints to support safety and effectiveness could help lower barriers to the implementation of novel MXR devices. This effort would benefit from collaboration among regulatory, research, and clinical communities.

#### Trial Controls

The development of methodologies for clinical trials using MXR devices poses unique challenges for trial design [35, 36]. To understand the clinical utility of MXR, comparative studies generally are needed to establish the incremental benefits and costs. For comparative studies intending to isolate the impact of MXR-based devices during medical procedures or therapies, there are a few options to consider for control conditions, including the best medical therapy, the standard of care, or therapy with a device or procedure that already has been approved for the indication of use and for controlled experimental designs. One controlled experimental approach is a crossover design in which the study participants serve as their own control group [37]. Although there are many considerations for determining the best control condition for a clinical trial, a sham control may be an appropriate option, especially for those device trials with subjective endpoints (e.g., pain). A sham control treatment or procedure is administered to ensure a participant experiences the same incidental effects as those who experience the true procedure or treatment. In these studies, a key treatment element is removed; for example, removing immersion by substituting 2D visualizations for 3D immersive environments. Use of a sham and rigorous blinding reduces potential confounding effects of bias from treatment allocation, treatment adherence, patient/user perceptions, and assessment of subjective outcomes modified by the treatment [37]. In the case of MXR device trials, designing a sham control and identifying the key therapeutic elements present additional challenges, since the interactions of a patient with a VR environment are not fully understood; including influence of the display type, content, and environment on outcomes. Also, the issue of blinding may be difficult to address. As research and development in the use of MXR devices continues, the broader community would benefit from continued open discussions and research into the best methods for MXR clinical trial designs. Table 2 summarizes the clinical evaluation challenges and examples of some of the remaining clinical questions for implementing MXR devices.

**Table 2:** Summary table of clinical evaluation challenges for MXR applications

**Trail Endpoints**	**Trial Controls**
Variety in intended use	Comparison to standard of care
Patient/clinician reported outcomes	Sham design
Performance outcome metrics	Patient blinding procedure

**Remaining Clinical Questions**	
Impact of device performance on outcomes	
Performance comparison to standard of care (SOC)	
Adjunct to SOC vs primary device	
Task-specific performance metrics	
Recommended frequency and duration of use (for therapeutic applications)	
Duration of therapeutic effects (for therapeutic applications)	

## Findings

The significance of these evaluation challenges heavily depends on the use case for a device. Table 3 shows examples of MXR Devices with FDA marketing authorization, which illustrates both the diversity in medical applications and the growing need to address key evaluation challenges. The classification product codes in Table 3 are used for classifying and tracking medical devices within CDRH. For example, the pertinent evaluation questions and performance requirements for a therapeutic application and a diagnostic task may differ, even if the applications use exactly the same hardware. In this section, we provide several examples illustrating the connection between the task, the indications for use, and the relevant evaluation challenges.

**Table 3:** Examples of MXR Devices with FDA Marketing Authorization

Submission Number	Applicant	Product Code	Specialty
K152915	SyncThink, Inc.	GWN	Neurology
K170793	Surgical Theater	LLZ	Radiology
K172418	Novarad	LLZ	Radiology
K183296	Penumbra	ISD	Phys. Medicine
K190764	MedVis	LLZ	Radiology
K190929	Augmedics	OLO	Orthopedic
K191014	Brainlab AG	LLZ	Radiology
K192890	SentiAR	LLZ	Cardiovascular
K193559	Medacta Intern.	PBF (JWH,OLO)	Orthopedic
K200384	Surgical Planning Assoc.	OSF	Orthopedic
K210072	RealView Imaging	LLZ	Radiology
K211254	Surgalign Spine Tech.	OLO (LLZ)	Orthopedic
DEN210005	Luminopia	QQU	Ophthalmic
DEN210014	AppliedVR	QRA	Phys. Medicine

### Image-guided Interventional and Surgical Applications

An important application space with significant evaluation challenges discussed at the public workshop was the use of AR devices in interventional procedures and surgery. The use cases for AR in the interventional procedures and surgery range from medical image visualization using a heads-up display with pertinent patient information such as vitals, to 3D visually guided interventional procedures, and the possibility of providing telementoring. Even with similarities in the environment and the AR hardware, evaluation challenges differ depending on a device’s intended use. For example, the safety and effectiveness concerns differ when a surgeon steps away from the patient to review preoperative medical images, versus when the surgeon is actively relying on AR during the procedure (real-time guidance). Additionally, the importance of image quality is relevant across AR interventional procedures and surgical applications, but the specifics of the indications for use determine the required image quality, as well as whether qualitative visual tests, quantitative bench tests, and/or clinical testing are necessary. Evaluation challenges for AR devices include visual and bench testing methods, resolution and contrast for 3D images, and performance thresholds for surgical tasks. In some cases, the safety risks of these new technologies can be mitigated by using the AR HMD as an adjunct display while maintaining the standard of care. MXR devices are also being explored as a mechanism to introduce image-guidance into procedures that are currently conducted bedside or in emergencies without medical images, such as catheter placement in external ventricular drainage procedures and have shown accuracy improvements in the catheter placement compared to the standard of care [33, 38, 39]. The addition of image-guidance into these bedside procedures also raises new questions regarding the required image quality, tracking, usability, and integration into the clinical workflow. AR-guided applications have additional evaluation challenges due to real-time tool tracking, frequently accomplished by combining an AR device with stereotaxics. AR combined with tool tracking raises additional evaluation challenges, such as measuring latency and the impact of latency on performance, spatial tool tracking accuracy, 3D registration accuracy of the tool relative to the patient and/or medical images, 3D registration accuracy of virtual models to patients, the stability of the registered images as the surgeon moves relative to the patient, and the impact of VAC on surgeon performance and depth perception. It is essential that the evaluation method matches the intended use to ensure the safety and effectiveness of the device for the application. The evaluation also should be minimally burdensome, in order to avoid creating unnecessary barriers to the adoption of safe and effective technologies.

### Clinical and Therapeutic Applications

MXR systems are being studied for their utility in addressing clinical indications in the field of behavioral medicine, including autism, post-traumatic stress, attention deficit hyperactivity disorder, substance abuse, depression, and other therapeutic applications [40–42]. One area of focus during the public workshop was VR for the management of pain. This emerging technology is a promising alternative or adjunct to opioids, and developments in this space have been supported by such government-sponsored programs as the Helping to End Addiction Long-Term (HEAL) initiative from the National Institute on Drug Abuse and the National Institutes of Health. Discussions in this area can readily guide current and future regulatory processes and the quality of evidence presented as part of device submissions.

The use of therapeutic VR presents challenges and considerations for clinical trial design and indications for use. One challenge is the development of a sham that provides patient blinding and isolates a therapeutic effect [43, 44], given the significant differences in the user interface compared to the standard of care. Recently developed studies are targeting engagement as the therapeutic element and comparing a minimally engaging 2D HMD-based sham to an immersive 3D HMD-based treatment [45–47]. Alternatively, studies have used a control condition such as the standard of care or a 2D monitor-based treatment [6]. The choice of a sham or control has implications for demonstrating effectiveness based on outcome measures. Additional considerations for condition-specific patient-reported outcomes include quality of life and function.

Another challenge is the choice of indications for use and the selection of outcome measures to support related claims. Regarding outcome measures, selection of appropriate metrics and elimination of confounding factors is challenging due to the multifactorial nature of pain. It also is common to assess function and quality of life in addition to perceived pain, but potential outcome measures can be difficult to interpret, or may not have been validated for the specific disease or population. Physiological signals and biomarkers for pain have similar limitations. Last, potentially important outcome measures may not have established criteria for meaningful change. For example, while the primary outcome of VR treatment is pain reduction, reduced opioid use is an attractive additional outcome for which clinically significant reduction has not yet been defined. Future work must address these challenges and explore other variables, such as frequency and duration of treatment, duration of therapeutic effects, types of VR content, user preferences and engagement, and efficacy across pain disorders.

## Call to Action

The consensus from the public workshop is that significant evaluation challenges for MXR devices persist across use cases. These can be categorized into a variety of technical and clinical challenges, which were summarized in this consensus article. To address these evaluation gaps, additional research is needed to characterize the performance of these devices from technical and medical performance perspectives. The relevant evaluation challenges and the specific assessment gaps primarily are determined by the intended use of a device. Therefore, development of suitable evaluation methods necessitates expertise across the MXR landscape in a precompetitive space to address the needs of the larger community. A number of potential avenues currently being explored would create the needed platforms for collaboration, including proposals to further the research establishing partnerships among industry, academia, and regulators. One current community effort to address these gaps and develop a framework for the addressing the evaluation gaps and challenges for MXR devices through the Medical Device Innovation Consortium (MDIC). Sustained community collaboration in a precompetitive space is necessary for the continued development of safe and effective MXR devices across medical applications.

## References

[1] Gupta, A., Ruijters, D., Flexman, M.L.: Augmented reality for interventional procedures. In: Digital Surgery, pp. 233–246. Springer (2020)

[2] Jud L, Fotouhi J, Andronic O, Aichmair A, Osgood G, Navab N, Farshad M (2020). Applicability of augmented reality in orthopedic surgery-a systematic review. BMC Musculoskeletal Disorders.

[3] Southworth MK, Silva JNA, Blume WM, Van Hare GF, Dalal AS, Silva JR (2020). Performance evaluation of mixed reality display for guidance during transcatheter cardiac mapping and ablation. IEEE Journal of Translational Engineering in Health and Medicine.

[4] Huang EY, Knight S, Guetter CR, Davis CH, Moller M, Slama E, Crandall M (2019). Telemedicine and telementoring in the surgical specialties: a narrative review. The American Journal of Surgery.

[5] Cherkin DC, Sherman KJ, Balderson BH, Cook AJ, Anderson ML, Hawkes RJ, Hansen KE, Turner JA (2016). Effect of mindfulness-based stress reduction vs cognitive behavioral therapy or usual care on back pain and functional limitations in adults with chronic low back pain: a randomized clinical trial. JAMA.

[6] Spiegel B, Fuller G, Lopez M, Dupuy T, Noah B, Howard A, Albert M, Tashjian V, Lam R, Ahn J (2019). Virtual reality for management of pain in hospitalized patients: A randomized comparative effectiveness trial. PlOS One.

[7] Carl E, Stein AT, Levihn-Coon A, Pogue JR, Rothbaum B, Emmelkamp P, Asmundson GJ, Carlbring P, Powers MB (2019). Virtual reality exposure therapy for anxiety and related disorders: A meta-analysis of randomized controlled trials. Journal of Anxiety Disorders.

[8] Kern, F., Winter, C., Gall, D., Käthner, I., Pauli, P., Latoschik, M.E.: Immersive virtual reality and gamification within procedurally generated environments to increase motivation during gait rehabilitation. In: 2019 IEEE Conference on Virtual Reality and 3D User Interfaces (VR), pp. 500–509. IEEE (2019)

[9] Beams, R., Collins, B., Kim, A.S., Badano, A.: Angular dependence of the spatial resolution in virtual reality displays. In: 2020 IEEE Conference on Virtual Reality and 3D User Interfaces (VR), pp. 836–841. IEEE (2020)

[10] Beams R, Kim AS, Badano A (2019). Transverse chromatic aberration in virtual reality head-mounted displays. Optics express.

[11] Hua H (2017). Enabling focus cues in head-mounted displays. Proceedings of the IEEE.

[12] Wilson A, Hua H (2017). Design and prototype of an augmented reality display with per-pixel mutual occlusion capability. Optics Express.

[13] Penczek J, Boynton PA, Meyer FM, Heft EL, Austin RL, Lianza TA, Leibfried LV, Gacy LW (2017). Absolute radiometric and photometric measurements of near-eye displays. Journal of the Society for Information Display.

[14] Aga, H., Ishihara, A., Kawasaki, K., Nishibe, M., Kohara, S., Ohara, T., Fukuchi, M.: 24-2: Latency compensation for optical see-through head-mounted with scanned display. In: SID Symposium Digest of Technical Papers, vol. 50, pp. 330–333. Wiley Online Library (2019)

[15] Mukawa, H.: Latency compensation for optical see-through ar headsets (conference presentation). In: Optical Architectures for Displays and Sensing in Augmented, Virtual, and Mixed Reality (AR, VR, MR), vol. 11310, p. 113101W. International Society for Optics and Photonics (2020)

[16] Kim AS, Cheng WC, Beams R, Badano A (2021). Color rendering in medical extended-reality applications. Journal of Digital Imaging.

[17] Olade I, Fleming C, Liang HN (2020). Biomove: Biometric user identification from human kinesiological movements for virtual reality systems. Sensors.

[18] IEC 63145-20-10:2019 Eyewear display - Part 20-10: Fundamental measurement methods - Optical properties (2020)

[19] IEC 63145-20-20:2019 Eyewear display - Part 20-20: Fundamental measurement methods - Image quality (2020)

[20] IEC 63145-22-10:2020 Eyewear display - Part 22-10: Specific measurement methods for AR type - Optical properties (2020)

[21] ISO 9241-303:2008 ergonomics of human-system interaction — Part 303: Requirements for electronic visual displays (2008)

[22] ISO 9241-392:2015 ergonomics of human-system interaction — part 392: Ergonomic recommendations for the reduction of visual fatigue from stereoscopic images (2015)

[23] Sun, X., Varshney, A.: Investigating perception time in the far peripheral vision for virtual and augmented reality. In: Proceedings of the 15th ACM Symposium on Applied Perception, pp. 1–8 (2018)

[24] Kress, B.C.: Digital optical elements and technologies (edo19): applications to AR/VR/MR. In: Digital Optical Technologies 2019, vol. 11062, p. 1106222. International Society for Optics and Photonics (2019)

[25] Carbone, M., Piazza, R., Condino, S.: Commercially available head-mounted displays are unsuitable for augmented reality surgical guidance: a call for focused research for surgical applications (2020) 10.1177/1553350620903197 32037972

[26] Condino S, Carbone M, Piazza R, Ferrari M, Ferrari V (2019). Perceptual limits of optical see-through visors for augmented reality guidance of manual tasks. IEEE Transactions on Biomedical Engineering.

[27] Hua, H.: Optical methods for enabling focus cues in head-mounted displays for virtual and augmented reality. In: Three-Dimensional Imaging, Visualization, and Display 2017, vol. 10219, p. 102190L. International Society for Optics and Photonics (2017)

[28] Leuze, C., Sathyanarayana, S., Daniel, B.L., McNab, J.A.: Landmark-based mixed-reality perceptual alignment of medical imaging data and accuracy validation in living subjects. In: 2020 IEEE International Symposium on Mixed and Augmented Reality (ISMAR), pp. 846–851. IEEE (2020)

[29] 21 CFR 820. 30 quality system regulation: Subpart C - design controls (2019) (2019)

[30] Baumeister J, Ssin SY, ElSayed NA, Dorrian J, Webb DP, Walsh JA, Simon TM, Irlitti A, Smith RT, Kohler M (2017). Cognitive cost of using augmented reality displays. IEEE transactions on visualization and computer graphics.

[31] Leuze, C., Yang, G., Hargreaves, B., Daniel, B., McNab, J.A.: Mixed-reality guidance for brain stimulation treatment of depression. In: 2018 IEEE International Symposium on Mixed and Augmented Reality Adjunct (ISMAR-Adjunct), pp. 377–380. IEEE (2018)

[32] Sathyanarayana, S., Leuze, C., Hargreaves, B., Daniel, B., Wetzstein, G., Etkin, A., Bhati, M.T., McNab, J.A.: Comparison of head pose tracking methods for mixed-reality neuronavigation for transcranial magnetic stimulation. In: Medical Imaging 2020: Image-Guided Procedures, Robotic Interventions, and Modeling, vol. 11315, p. 113150L. International Society for Optics and Photonics (2020)

[33] Sun X, Murthi SB, Schwartzbauer G, Varshney A (2020). High-precision 5DoF tracking and visualization of catheter placement in EVD of the brain using AR. ACM Transactions on Computing for Healthcare.

[34] Pratt, P., Ives, M., Lawton, G., Simmons, J., Radev, N., Spyropoulou, L., Amiras, D.: Through the hololens^™^ looking glass: augmented reality for extremity reconstruction surgery using 3d vascular models with perforating vessels. European Radiology Experimental **2**(1), 2 (2018) 10.1186/s41747-017-0033-2 PMC5909360 29708204

[35] Birckhead B, Khalil C, Liu X, Conovitz S, Rizzo A, Danovitch I, Bullock K, Spiegel B (2019). Recommendations for methodology of virtual reality clinical trials in health care by an international working group: iterative study. JMIR Mental Health.

[36] Dascal J, Reid M, IsHak WW, Spiegel B, Recacho J, Rosen B, Danovitch I (2017). Virtual reality and medical inpatients: A systematic review of randomized, controlled trials. Innovations in Clinical Neuroscience.

[37] Sutherland ER (2007). Sham procedure versus usual care as the control in clinical trials of devices: which is better?. Proceedings of the American Thoracic Society.

[38] Azimi, E., Niu, Z., Stiber, M., Greene, N., Liu, R., Molina, C., Huang, J., Huang, C.M., Kazanzides, P.: An interactive mixed reality platform for bedside surgical procedures. In: International Conference on Medical Image Computing and Computer-Assisted Intervention, pp. 65–75. Springer (2020)

[39] Li Y, Chen X, Wang N, Zhang W, Li D, Zhang L, Qu X, Cheng W, Xu Y, Chen W (2018). A wearable mixed-reality holographic computer for guiding external ventricular drain insertion at the bedside. Journal of neurosurgery.

[40] Jerdan SW, Grindle M, van Woerden HC, Boulos MNK (2018). Head-mounted virtual reality and mental health: critical review of current research. JMIR Serious Games.

[41] Maggio MG, De Luca R, Molonia F, Porcari B, Destro M, Casella C, Salvati R, Bramanti P, Calabro RS (2019). Cognitive rehabilitation in patients with traumatic brain injury: A narrative review on the emerging use of virtual reality. Journal of Clinical Neuroscience.

[42] Spiegel, B.: VRx: How Virtual Therapeutics Will Revolutionize Medicine. Station Hill Press, Inc. (2020)

[43] Colloca L, Raghuraman N, Wang Y, Akintola T, Brawn-Cinani B, Colloca G, Kier C, Varshney A, Murthi S (2020). Virtual reality: physiological and behavioral mechanisms to increase individual pain tolerance limits. Pain.

[44] Honzel E, Murthi S, Brawn-Cinani B, Colloca G, Kier C, Varshney A, Colloca L (2019). Virtual reality, music, and pain: developing the premise for an interdisciplinary approach to pain management. Pain.

[45] Garcia, L.M., Birckhead, B.J., Krishnamurthy, P., Mackey, I., Sackman, J., Salmasi, V., Louis, R., Maddox, T., Darnall, B.D.: Three-month follow-up results of a double-blind, randomized placebo-controlled trial of 8-week self-administered at-home behavioral skills-based virtual reality (vr) for chronic low back pain. The journal of pain (2021) 10.1016/j.jpain.2021.12.002 34902548

[46] Garcia LM, Birckhead BJ, Krishnamurthy P, Sackman J, Mackey IG, Louis RG, Salmasi V, Maddox T, Darnall BD (2021). An 8-week self-administered at-home behavioral skills-based virtual reality program for chronic low back pain: Double-blind, randomized, placebo-controlled trial conducted during covid-19. J Med Internet Res.

[47] Spiegel, B.: Randomized-controlled trial of virtual reality for chronic low back pain to improve patient-reported outcomes and physical activity (2020)

